# Are the *Culex pipiens* biotypes *pipiens*, *molestus* and their hybrids competent vectors of avian *Plasmodium*?

**DOI:** 10.1371/journal.pone.0314633

**Published:** 2024-12-03

**Authors:** Rafael Gutiérrez-López, Jiayue Yan, Laura Gangoso, Ramón Soriguer, Jordi Figuerola, Josué Martínez-de la Puente

**Affiliations:** 1 National Center of Microbiology, Health Institute Carlos III, Madrid, Spain; 2 CIBER de Enfermedades Infecciosas (CIBERINFEC), Madrid, Spain; 3 Illinois Natural History Survey, University of Illinois at Urbana-Champaign, Champaign, IL, United States of America; 4 Department of Biodiversity, Ecology and Evolution, Faculty of Biological Sciences, Complutense University of Madrid, Madrid, Spain; 5 Doñana Biological Station, Seville, Spain; 6 CIBER Epidemiología y Salud Pública (CIBERESP), Madrid, Spain; University of Agricultural Sciences and Veterinary Medicine Cluj-Napoca, Life Science Institute, ROMANIA

## Abstract

The common house mosquito *Culex pipiens* s.l., widely distributed in Europe, Africa, and North America has two recognized biotypes, *Cx*. *pipiens* biotype *pipiens* and *Cx*. *pipiens* biotype *molestus*, which hybridize. Despite their morphological similarities, these biotypes may exhibit ecological differences. This complex ecological mosaic may affect the interaction of *Cx*. *pipiens* with pathogens like avian *Plasmodium*, which is transmitted to wildlife. Although the vector competence for *Cx*. *pipiens* biotype *molestus* has been well studied, there is a lack of studies comparing the vector competence of *Cx*. *pipiens* biotype *pipiens* and their hybrids for the transmission of avian *Plasmodium*. Here, we evaluated the vector competence of the *Cx*. *pipiens* biotypes *pipiens*, *molestus* and their hybrids for the transmission of two avian *Plasmodium* species. Mosquitoes were allowed to feed on blood of wild infected birds and the presence of DNA of *Plasmodium* in head-thorax and saliva of mosquitoes was molecularly evaluated at 13 day-post exposure. The transmission rates (i.e., the detection of parasite DNA in mosquito saliva) for *Plasmodium cathemerium* were similar for the two biotypes of *Cx*. *pipiens* and their hybrids while *Plasmodium relictum* DNA was only found in the saliva of *Cx*. *pipiens* biotype *pipiens*. In addition, *P*. *cathemerium* was significantly more prevalent than *P*. *relictum* in the saliva of *Cx*. *pipiens* biotype *pipiens*. Our results suggest that avian *Plasmodium* is transmitted by both *Cx*. *pipiens* biotypes and their hybrids although differences could be found depending of the parasite species studied. Differences in the abundance of each biotype and their hybrids within areas characterized by distinct environmental conditions, along with variations in their blood-feeding patterns and the parasites infecting birds, could ultimately determine differences in the relevance of each *Cx*. *pipiens* biotype in the transmission of avian *Plasmodium*.

## Introduction

The common house mosquito *Culex pipiens* s.l. is widely distributed in temperate regions across the northern hemisphere, including Europe and Africa and, more recently, America, Asia, and Australia [1, 2], where it usually breeds in anthropized habitats. In Europe, the *Culex pipiens* complex (*Cx*. *pipiens* s.l.) consists of several species, including *Culex pipiens* s.s. (Linnaeus, 1758) and *Culex quinquefasciatus* (Say, 1823) [3]. In addition, for *Cx*. *pipiens* s.s., two biotypes are recognized, namely *Cx*. *pipiens* biotype *pipiens* and *Cx*. *pipiens* biotype *molestus*. The *Cx*. *pipiens* biotype *pipiens* is typically associated with aboveground habitats, such as standing water in clogged gutters or artificial containers, and may exhibit a bird-biased blood-feeding pattern [4, 5, but see 6]. In contrast, *Cx*. *pipiens* biotype *molestus* (Forskål, 1775) is frequently found in underground habitats, such as basements, tunnels, and sewers. The *Cx*. *pipiens* biotype *molestus* tends to feed on mammalian blood, including humans as hosts, and exhibits autogeny, that is, the ability to produce eggs without a blood meal [7]. Nevertheless, the classification of *Cx*. *pipiens* biotype *pipiens* as strictly ornithophilic and the *Cx*. *pipiens* biotype *molestus* as mammalophilic may be considered simplistic, as both *Cx*. *pipiens* biotypes feed on a variety of birds and mammals [6]. These two *Cx*. *pipiens* biotypes are capable of interbreeding, and hybrids are suggested to exhibit intermediate host-feeding behavior, also incorporating both mammals and birds into their diet [2–4, 7]. This complex ecological mosaic may influence the ability of mosquitoes to interact with pathogens that infect different vertebrate groups, potentially affecting their transmission. For instance, the *Cx*. *pipiens* biotype *pipiens* could play an important role in the natural transmission cycle of West Nile virus (WNV) among birds, while *Cx*. *pipiens* hybrids have been proposed as more suitable bridge vectors of WNV from birds to humans [7].

Avian *Plasmodium* is a mosquito-borne haemosporidian parasite with significant impacts on both livestock and wildlife [8]. These parasites are considered as model pathogens for studying the ecology and evolution of vector-pathogen interactions [9], and share some characteristics, including the insect vectors, with other avian pathogens such as WNV [10]. Sporozoites, the infective forms of avian *Plasmodium*, accumulate in the mosquito’s salivary glands and are transmitted to the bloodstream of new bird hosts through mosquito bites. Several mosquito species are involved in transmitting malarial parasites, with species of the *Culex* genus playing a key role as vectors of avian *Plasmodium spp*. [11]. *Culex pipiens* has been experimentally confirmed as a vector of different *Plasmodium* species [11–13].

In southern Europe, including Spain, both *Cx*. *pipiens* biotype *pipiens* and *molestus* and their hybrids are frequently found [2], where they exhibit similar feeding patterns, which promotes frequent interactions with avian *Plasmodium* under natural conditions [14]. Different studies have experimentally demonstrated the transmission capacity of both *Cx*. *pipiens* biotypes for several avian *Plasmodium* species. For instance, *Plasmodium relictum* is known to complete sporogony in both *Cx*. *pipiens* biotypes [11, 15–17]. Vector competence reflects the intrinsic ability of an arthropod vector to transmit an infectious agent through its bites [18]. Although, most studies have been conducted using *Cx*. *pipiens* biotype *molestus*, probably favored by its ability to breed in colonies, there is a lack of studies comparing the vector competence between *Cx*. *pipiens* biotypes and their hybrids for the transmission of avian *Plasmodium*. Thus, our aim is to experimentally assess the vector competence of *Cx*. *pipiens* biotype *pipiens* and *Cx*. *pipiens* biotype *molestus* as well as their hybrids for the transmission of two avian *Plasmodium* species, namely *P*. *relictum* and *Plasmodium cathemerium* using the forced salivation technique [19].

## Materials and methods

### Mosquito collection and rearing conditions

Mosquitoes analysed in Gutiérrez-López et al. [16] were used in this study. Here, we molecularly determined the biotype of the *Cx*. *pipiens* mosquitoes to compare the vector competence of the different *Cx*. *pipiens* biotypes for avian *Plasmodium* transmission. *Culex pipiens* larvae were collected in La Cañada de los Pájaros, Andalusia, Spain (6°14′W, 36°57′N) during summer 2014. Mosquito larvae were transported to the facilities of the Estación Biologica de Doñana (EBD-CSIC) where they were maintained in a climatic chamber at 28°C, 65–70% RH and 12:12 light: dark cycle [19]. Adult female mosquitoes were identified to the species level following Schaffner et al. [20] and placed in insect cages (BugDorm-43030F, 32.5 × 32.5 × 32.5 cm). Mosquitoes were fed *ad libitum* with 1% sugar solution. Two-three-week-old female mosquitoes were deprived of the sugar solution one day prior to each experimental exposure to birds.

### Bird sampling and maintenance

Three juvenile house sparrows (*Passer domesticus*) were captured using mist nets in the Huelva province. The birds were individually ringed with a numbered metal ring. The birds were blood sampled from the jugular vein. A drop of blood was smeared to quantify their parasitaemia [21] and, subsequently, the intensity of infection by *Plasmodium* and *Haemoproteus* parasites was estimated as the percentage of infected cells per 100 erythrocytes after counting 4,000–10,000 erythrocytes at ×1000 magnification. The birds were transported to the Unit of Animal Experimentation at the EBD-CSIC and maintained in birdcages (58.5×25×36 cm) in a vector-free room under controlled conditions (23 ± 1°C, 40–50% RH and 12:12 light: dark cycle). The birds had *ad libitum* access to a standard mixed diet for seed-eating and insectivorous birds (KIK, GZM S.L., Alicante, Spain). Three days after exposure to mosquitoes, the birds were blood-sampled again (0.2 ml) to detect any potential change in infection status or parasite lineage identity. No changes were observed during the experiment. The birds were released at the site of capture at the end of the experiment.

### Exposure procedure

After 11 days of acclimation, each bird was individually housed in a birdcage (38.5×25.5×26 cm) placed within an insect tent (BugDorm-2120, 60×60×60 cm). Over the course of four separate nights, each bird was introduced into independent tents and exposed to unfed *Culex pipiens* females. The number of mosquitoes used per bird each night varied according to the availability of 2-3-week-old unfed mosquitoes: 50 on the first night, 57 on the second night, 105 on the third night, and 100 on the fourth night (overall, 312 mosquitoes were allowed to feed on each bird). Birds were exposed to mosquitoes from 8:00 pm to 8:00 am. Mosquitoes were only exposed to birds once. After each exposure, mosquitoes with visible blood meals were separated and kept in insect cages (BugDorm-43030F) under the previously mentioned conditions during 13 days. Previous studies found that avian *Plasmodium* complete its development in mosquitoes in 8–13 days [8, 22]. During this period, engorged mosquitoes had *ad libitum* access to 1% sugar solution. At 13 days post exposure (dpe), we extracted the saliva of mosquitoes following ref. [19]. Subsequently, mosquitoes were dissected and the head-thorax portion containing the salivary glands, was stored individually in Eppendorf tubes. Due to logistical problems, the head-thoraxes and saliva of seven mosquitoes and the saliva of two additional mosquitoes that survived until 13 dpe were not examined. All samples were kept at −80°C until further molecular analyses. Regional authorities (Junta de Andalucía) and the CSIC Ethics Committee approved the procedures used in this study (ref. CEBA-EBD-12-40).

### Molecular identification of blood parasites and mosquito biotypes

We used the MAXWELL® 16 LEV Blood DNA Kit for the extraction of genomic DNA from birds’ blood samples (both the initial and final samples) following [23]. DNA from the head-thorax and saliva of mosquitoes was extracted using the Qiagen DNeasy® Kit Tissue and Blood (Qiagen, Hilden, Germany). A 478-bp fragment of the mitochondrial cytochrome b gene of *Plasmodium / Haemoproteus* parasites was amplified using the procedure described in [24]. DNA extracted from the head-thorax of mosquitoes analysed for the presence of *Plasmodium* parasites was used to identify the *Cx*. *pipiens* biotype by amplifying the 5′ flacking region of CQ11 microsatellite [25]. Amplicons were verified on 2% agarose gels and sequenced in both directions with the Macrogen sequencing service (Macrogen Inc., Amsterdam, The Netherlands). Parasite sequences were edited using the Sequencher™ software v 4.9 (Gene Codes Corp. © 1991–2009, Ann Arbor, MI 48108). Parasite lineages and morphospecies were identified by blast comparison with sequences deposited in GenBank (National Center for Biotechnology Information) and Malavi [26].

### Statistical analysis

The presence of parasite DNA in the head-thorax of mosquitoes was used as an indicator of the infection rate. Infection rate was calculated as the number of *Plasmodium* positive mosquitoes divided by the number of blood-fed females analysed. The presence of parasite DNA in mosquito saliva was used as an indicator of the transmission rate and was calculated as the number of mosquitoes with *Plasmodium* positive saliva divided by the number of mosquitoes with *Plasmodium* positive head-thoraxes. We tested for differences in *Plasmodium* infection and transmission rates among both *Culex* biotypes and their hybrids using Generalized Linear Mixed Models GLMMs with binomial error and a logit link function. The *Cx*. *pipiens* biotype was included as a factor with three levels (*pipiens*, *molestus* and hybrids) while bird identity was included as a random term. In addition, we assessed the effect of *Plasmodium* species on the infection status by including the parasite identity as the only predictor variable in the former models. This model only was done for *Cx*. *pipiens* biotype *pipiens* due to the absence of *P*. *relictum* in the saliva of *Cx*. *pipiens* biotype *molestus* and hybrids (see results). We conducted a third model to test differences in *P*. *cathemerium* infection in the head-thorax and saliva of mosquitoes from both *Cx*. *pipiens* biotypes and their hybrids. Fixed effects were tested using likelihood ratio tests. Overdispersion was checked using the overdispFun function. Statistical analyses were conducted using the package lme4 [27] in R version 4.3.2 (R Core Development Team, 2016) [28].

## Results

Among the three bird donors, one individual (house sparrow 1) was infected with the *P*. *relictum* lineage GRW11 (parasite load <0.001%), another bird (house sparrow 2) was infected with the *P*. *cathemerium* lineage PADOM01 (parasite load = 0.2%), and the third bird (house sparrow 3) was infected with the *P*. *relictum* lineage SGS1 (parasite load = 0.3%). All birds also exhibited coinfection with *Haemoproteus* PADOM05, a parasite transmitted by *Culicoides* spp. but not by mosquitoes.

Overall, 126 out of 936 mosquitoes (13.5%) used in this study fed on bird blood, with 105 individuals surviving until 13 days post-exposure (dpe). A total of 98 head-thorax and 96 saliva samples were analysed molecularly. Among these, 52 corresponded to *Cx*. *pipiens* biotype *pipiens* (40 exposed to birds infected with *P*. *relictum* and 12 exposed to a bird infected with *P*. *cathemerium*), 7 corresponded to *Cx*. *pipiens* biotype *molestus* (5 exposed to *P*. *relictum* and 2 to *P*. *cathemerium*), and 39 corresponded to hybrids (27 exposed to *P*. *relictum* and 12 to *P*. *cathemerium*) (Table 1 and Table 2).

**Table 1:** Head-thorax and saliva analysed from the different *Cx*. *pipiens* biotypes exposed to *Plasmodium* species.

*Cx*. *pipiens* biotype	*Plasmodium* exposure	Head-thorax positive/analysed	Infection rate (%)	Saliva positive/analysed	Transmission rate (%)
*Pipiens*	*P*. *relictum*	13/40	32.5	1/11	9.1
	*P*. *cathemerium*	10/12	83.3	4/10	40.0
*Molestus*	*P*. *relictum*	1/5	20	0/1	0
	*P*. *cathemerium*	2/2	100	1/2	50.0
Hybrids	*P*. *relictum*	7/27	25.9	0/7	0
	*P*. *cathemerium*	11/12	91.7	2/11	18.2

**Table 2:** Number of *Culex pipiens* biotype *pipiens*, *molestus* and hybrids analyzed from the mosquitoes exposed to the donor birds infected with avian *Plasmodium*.

Bird donor	*Plasmodium* infection in birds	*Cx*. *pipiens* biotype *pipiens*	*Cx*. *pipiens* biotype *molestus*	*Cx*. *pipiens* hybrids
House sparrow 1	*P*. *relictum* (lineage GRW11)	21	4	12
House sparrow 2	*P*. *cathemerium* (lineage PADOM01)	12	2	11
House sparrow 3	*P*. *relictum* (lineage SGS1)	19	1	16

Among the mosquitoes analysed, 23 *Cx*. *pipiens* biotype *pipiens* mosquitoes (44.2%; N = 52) tested positive for *Plasmodium* in the head-thorax. Of these, five individuals (21.7%) also had *Plasmodium* DNA in their saliva. Three *Cx*. *pipiens* biotype *molestus* mosquitoes (42.9%; N = 7) had *Plasmodium* DNA in the head-thorax, with only one individual (33.4%) also showing *Plasmodium* DNA in the saliva. Eighteen hybrids (46.2%; N = 39) were positive for *Plasmodium* DNA in the head-thorax, with two of them (11.1%) also showing positive results in their saliva.

*Culex pipiens* biotype *pipiens* was able to transmit *P*. *relictum* and *P*. *cathemerium*, as supported by the detection of DNA of both *Plasmodium* species in their head-thorax and saliva (Table 1). *Cx*. *pipiens* biotype *molestus* tested positive for *P*. *cathemerium* in their head-thorax and saliva, but *P*. *relictum* DNA was only detected in the head-thorax of mosquitoes (Table 1). In addition, *P*. *relictum* DNA was found in the head-thorax of *Cx*. *pipiens* hybrids, but it was not detected in the mosquito saliva, meanwhile *P*. *cathemerium* DNA was present in both the head-thorax and saliva of hybrids (Table 1). Table 3 summarized the number of mosquitoes analysed exposed to each bird.

**Table 3:** Infection status of bird donors, number of engorged and analysed mosquitoes.

Bird donor	*Plasmodium* infection in birds	Engorged mosquitoes	Alive mosquitoes at 13 dpe	Head-thorax positive/analysed	Saliva positive/analysed
House sparrow 1	*P*. *relictum* (lineage GRW11)	42 (13.5%)	36 (85.7%)	1/36	0/36
House sparrow 2	*P*. *cathemerium* (lineage PADOM01)	39 (12.5%)	33 (84.6%)	23/26	7/26
House sparrow 3	*P*. *relictum* (lineage SGS1)	45 (14.4%)	36 (80%)	20/36	1/34

**Fig 1:**
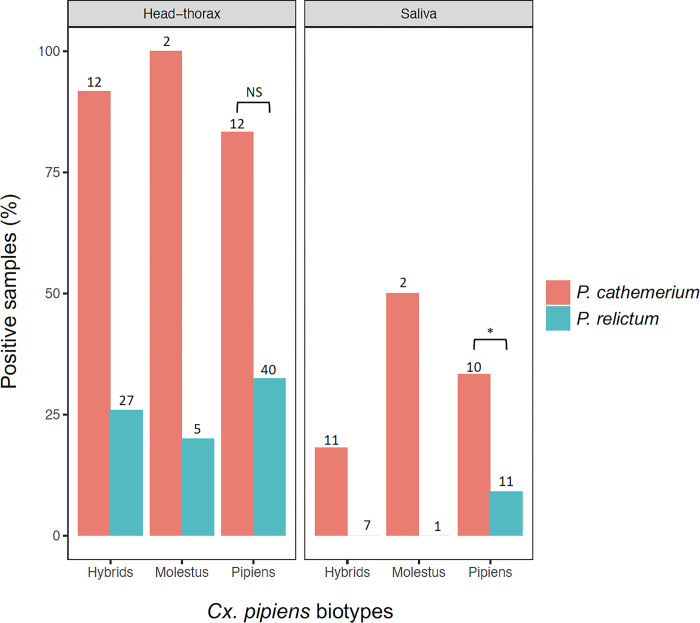
Positive samples for Plasmodium DNA in head-thoraxes and saliva from the different mosquito biotypes analysed for P. **relictum and P. cathemerium.** Sample size indicated over each bar. Statistically significant differences are indicated with an asterisk (*). NS means non-significant differences.

The two *Cx*. *pipiens* biotypes and their hybrids showed a similar *Plasmodium* prevalence in the head-thorax (χ2 = 1.25; d.f. = 2; P = 0.53). In addition, non-significant differences were found for the presence of *Plasmodium* DNA in the saliva of mosquitoes of the two biotypes and their hybrids (χ2 = 1.90; d.f. = 2; P = 0.39). We did not find significant differences in the prevalence of *P*. *cathemerium* in the head-thorax (χ2 = 0.90; d.f. = 2; P = 0.64) or in the saliva (χ2 = 1.43; d.f. = 2; P = 0.49) between *Cx*. *pipiens* biotypes and their hybrids, suggesting similar vector competence for *P*. *cathemerium*. Regarding the *Cx*. *pipiens* biotype *pipiens*, the prevalence of *Plasmodium* in the head-thorax was similar between both *Plasmodium* species (Fig 1; χ2 = 1.28; d.f. = 1; P = 0.26). However, significant differences were found for the prevalence of the two parasite species in the saliva of *Cx*. *pipiens* biotype *pipiens* (χ2 = 5.06; d.f. = 1; P = 0.02), being *P*. *cathemerium* more prevalent than *P*. *relictum* (40.0% and 9.1%, respectively) (Fig 1).

## Discussion

Previous studies on the competence of *Cx*. *pipiens* biotypes *pipiens* and *molestus* and their hybrids for transmitting avian pathogens have revealed potential differences between them. For instance, although all these mosquitoes can transmit WNV, differences arose depending on temperature [29, 30]. Here, using avian malaria parasites as study model, we provide evidence for a similar competence of the *Cx*. *pipiens* biotype *pipiens* and *Cx*. *pipiens* biotype *molestus* and their hybrids to transmit avian malaria parasites. However, we found differences between *Plasmodium* species in their capacity to develop in these mosquitoes.

Previous studies using *Cx*. *pipiens* colonies have observed that the widespread *P*. *relictum* lineages SGS1 and GRW11, can complete sexual reproduction in *Cx*. *pipiens* biotype *molestus*, with sporozoites, the infective forms, being detected in the salivary glands of female mosquitoes [15, 31]. Similarly, *P*. *relictum* lineage GRW4 completed sporogony in *Cx*. *pipiens* biotype *molestus*, with a considerable number of sporozoites being accumulated in the salivary glands [16]. Avian *Plasmodium* parasites, including *P*. *relictum*, have been found in *Cx*. *pipiens* biotype *pipiens*, *Cx*. *pipiens* biotype *molestus* and their hybrids [14, 32, 33]. In mosquitoes from Eastern Austria, the avian *Plasmodium* prevalence was 2.33% in *Cx*. *pipiens* biotype *molestus*, 1.99% in *Cx*. *pipiens* biotype *pipiens*, and 5.26% in the hybrids [33]. However, in this study, *P*. *relictum* was only found in *Cx*. *pipiens* biotype *pipiens* [33]. In mosquitoes from Japan [32], avian *Plasmodium* was also found in *Cx*. *pipiens* biotype *molestus* with a prevalence of 13.5%. These results support the potential role of *Cx*. *pipiens* biotype *molestus* in the transmission of avian *Plasmodium* under natural conditions.

Here, by exposing female mosquitoes emerged from field-collected larvae to naturally infected birds, we provide evidence for the transmission of *P*. *cathemerium* lineage PADOM01 in *Cx*. *pipiens* of the two biotypes, *pipiens* and *molestus*, and their hybrids with a similar prevalence between them. These results are especially relevant to confirm the vector competence of *Cx*. *pipiens* for avian *Plasmodium* since most studies used mosquitoes maintained in colonies. Vector competence may differ between wild-collected and colony-maintained mosquitoes, due to the higher likelihood of bottlenecks and genetic drift processes in the colonies [34–36], potentially influencing the conclusions drawn. We found significant differences in the transmission rate between *Plasmodium* species in *Cx*. *pipiens* biotype *pipiens*, being *P*. *cathemerium* more prevalent than *P*. *relictum* as previously reported [12], a pattern evident only when the presence of parasite DNA was tested in mosquito saliva.

Different factors could affect the vector competence of avian *Plasmodium* by the *Cx*. *pipiens* biotypes, including the time required for *Plasmodium* species to develop and reach the salivary glands (i.e., the extrinsic incubation period). *Plasmodium cathemerium* sporozoites have been identified in the salivary glands of *Cx*. *pipiens* at 7 dpe [37], while *P*. *relictum* sporozoites have been found as early as 4 and 5 dpe [38, 39]. However, in *Cx*. *pipiens*, sporozoites of *P*. *relictum* lineages SGS1 and GRW11 were not detected in the salivary glands until 14 dpe (the presence of sporozoites was verified at 12 dpe, but not at 13 dpe) [40].

The intensity of *Plasmodium* infection in birds could also have an effect on the success of parasite development within the vector and, consequently, in the ability to transmit the parasite. A positive relationship between the density of *Plasmodium* gametocytes and the proportion of oocysts in mosquitoes was found in humans [41]. However, this was not the case of a previous study using avian *Plasmodium* [42]. Similarly, the antimalarial treatment and the subsequent reduction of parasite load in birds did not affect the proportion of mosquitoes with *Plasmodium*-positive head-thoraxes or saliva [43]. These results suggest that avian *Plasmodium* may still develop in mosquitoes, even when vectors feed on birds with low infection intensities, undetectable by microscopy [41, 43]. Furthermore, mosquito age may also affect the parasite development, as older mosquitoes are more resistant to avian *Plasmodium* infections compared to younger ones [44]. In this study, 2- to 3-week-old mosquitoes were used, which could potentially reduce the observed infection prevalence. Nevertheless, we did not expect this factor to cause differences between *Cx*. *pipiens* biotypes and avian *Plasmodium* species, as mosquitoes were randomly distributed across the experiments. Further experimental studies are necessary to explore the impact of mosquito age on vector competence for avian *Plasmodium*.

In conclusion, *Cx pipiens* biotypes may have differential roles in the epidemiology of avian pathogens such as WNV [45]. Our results support that the competence of both *Cx*. *pipiens* biotypes and their hybrids to transmit avian *Plasmodium* may be similar, although differences may arise depending on the species of *Plasmodium* studied. Under an ecological perspective, the results reported here should be contextualized with data about the abundance of each *Cx*. *pipiens* biotype and their hybrids according to different landscape characteristics and other ecological factors which could affect their abundance in a particular area, including the different species of parasites circulating in the area [2, 14].

## Supporting information

S1 File (DOCX)
