# Supplementary material for: Are the Culex pipiens biotypes pipiens, molestus and their hybrids competent vectors of avian Plasmodium?
Source: PLoS One. 2024 Dec 3;19(12):e0314633. doi: 10.1371/journal.pone.0314633 (PMC11614271; doi:10.1371/journal.pone.0314633)
Supplement: S1 File — (DOCX) [file pone.0314633.s001.docx]

**Supplementary Material**

**Are the *Culex pipiens* biotypes *pipiens*, *molestus* and their hybrids competent vectors of avian *Plasmodium*?**

Rafael Gutiérrez-López^1,2^*; Jiayue Yan^3^; Laura Gangoso^4^; Ramón Soriguer^5,6^; Jordi Figuerola^5,6^; Josué Martínez-de la Puente^5,6^*

**Database used during the study**.

| **Mosquito identity** | **Infection Head-Thorax** | **Infection Saliva** | ***Cx. pipiens* biotype** | **Bird identity** | ***Plasmodium* lineage** | ***Plasmodium* species** |
| --- | --- | --- | --- | --- | --- | --- |
| 1 | 1 | 0 | Pipiens | House sparrow 3 | SGS1 | *Plasmodium relictum* |
| 2 | 1 | 0 | Pipiens | House sparrow 2 | PADOM01 | *Plasmodium cathemerium* |
| 3 | 1 | 0 | Hybrids | House sparrow 2 | PADOM01 | *Plasmodium cathemerium* |
| 4 | 1 | 1 | Molestus | House sparrow 2 | PADOM01 | *Plasmodium cathemerium* |
| 5 | 0 | 0 | Hybrids | House sparrow 1 | GRW11 | *Plasmodium relictum* |
| 6 | 0 | 0 | Pipiens | House sparrow 1 | GRW11 | *Plasmodium relictum* |
| 7 | 0 | 0 | Pipiens | House sparrow 1 | GRW11 | *Plasmodium relictum* |
| 8 | 0 | 0 | Hybrids | House sparrow 1 | GRW11 | *Plasmodium relictum* |
| 9 | 0 | 0 | Pipiens | House sparrow 1 | GRW11 | *Plasmodium relictum* |
| 10 | 0 | 0 | Pipiens | House sparrow 1 | GRW11 | *Plasmodium relictum* |
| 11 | 0 | 0 | Molestus | House sparrow 1 | GRW11 | *Plasmodium relictum* |
| 12 | 0 | 0 | Pipiens | House sparrow 1 | GRW11 | *Plasmodium relictum* |
| 13 | 1 | 0 | Hybrids | House sparrow 3 | SGS1 | *Plasmodium relictum* |
| 14 | 1 | 1 | Pipiens | House sparrow 3 | SGS1 | *Plasmodium relictum* |
| 15 | 1 | 0 | Hybrids | House sparrow 2 | PADOM01 | *Plasmodium cathemerium* |
| 16 | 1 | 0 | Pipiens | House sparrow 2 | PADOM01 | *Plasmodium cathemerium* |
| 17 | 1 | 0 | Hybrids | House sparrow 2 | PADOM01 | *Plasmodium cathemerium* |
| 18 | 1 | 0 | Hybrids | House sparrow 2 | PADOM01 | *Plasmodium cathemerium* |
| 19 | 0 | 0 | Hybrids | House sparrow 3 | SGS1 | *Plasmodium relictum* |
| 20 | 1 | 0 | Pipiens | House sparrow 3 | SGS1 | *Plasmodium relictum* |
| 21 | 0 | 0 | Hybrids | House sparrow 3 | SGS1 | *Plasmodium relictum* |
| 22 | 0 | 0 | Pipiens | House sparrow 3 | SGS1 | *Plasmodium relictum* |
| 23 | 0 | 0 | Pipiens | House sparrow 3 | SGS1 | *Plasmodium relictum* |
| 24 | 1 | 0 | Pipiens | House sparrow 3 | SGS1 | *Plasmodium relictum* |
| 25 | 0 | 0 | Hybrids | House sparrow 3 | SGS1 | *Plasmodium relictum* |
| 26 | 1 | 0 | Pipiens | House sparrow 3 | SGS1 | *Plasmodium relictum* |
| 27 | 1 | 0 | Pipiens | House sparrow 3 | SGS1 | *Plasmodium relictum* |
| 28 | 1 | 0 | Hybrids | House sparrow 3 | SGS1 | *Plasmodium relictum* |
| 29 | 1 | 0 | Pipiens | House sparrow 3 | SGS1 | *Plasmodium relictum* |
| 30 | 0 | 0 | Pipiens | House sparrow 1 | GRW11 | *Plasmodium relictum* |
| 31 | 0 | 0 | Hybrids | House sparrow 1 | GRW11 | *Plasmodium relictum* |
| 32 | 0 | 0 | Pipiens | House sparrow 1 | GRW11 | *Plasmodium relictum* |
| 33 | 0 | 0 | Pipiens | House sparrow 1 | GRW11 | *Plasmodium relictum* |
| 34 | 0 | 0 | Pipiens | House sparrow 1 | GRW11 | *Plasmodium relictum* |
| 35 | 0 | 0 | Hybrids | House sparrow 1 | GRW11 | *Plasmodium relictum* |
| 36 | 0 | 0 | Hybrids | House sparrow 1 | GRW11 | *Plasmodium relictum* |
| 37 | 0 | 0 | Pipiens | House sparrow 1 | GRW11 | *Plasmodium relictum* |
| 38 | 0 | 0 | Hybrids | House sparrow 1 | GRW11 | *Plasmodium relictum* |
| 39 | 0 | 0 | Pipiens | House sparrow 1 | GRW11 | *Plasmodium relictum* |
| 40 | 0 | 0 | Molestus | House sparrow 1 | GRW11 | *Plasmodium relictum* |
| 41 | 0 | 0 | Pipiens | House sparrow 1 | GRW11 | *Plasmodium relictum* |
| 42 | 0 | 0 | Pipiens | House sparrow 1 | GRW11 | *Plasmodium relictum* |
| 43 | 0 | 0 | Hybrids | House sparrow 1 | GRW11 | *Plasmodium relictum* |
| 44 | 0 | 0 | Molestus | House sparrow 1 | GRW11 | *Plasmodium relictum* |
| 45 | 0 | 0 | Pipiens | House sparrow 1 | GRW11 | *Plasmodium relictum* |
| 46 | 0 | 0 | Pipiens | House sparrow 1 | GRW11 | *Plasmodium relictum* |
| 47 | 1 | 0 | Hybrids | House sparrow 1 | GRW11 | *Plasmodium relictum* |
| 48 | 0 | 0 | Molestus | House sparrow 1 | GRW11 | *Plasmodium relictum* |
| 49 | 0 | 0 | Hybrids | House sparrow 1 | GRW11 | *Plasmodium relictum* |
| 50 | 0 | 0 | Pipiens | House sparrow 1 | GRW11 | *Plasmodium relictum* |
| 51 | 0 | 0 | Hybrids | House sparrow 1 | GRW11 | *Plasmodium relictum* |
| 52 | 0 | 0 | Pipiens | House sparrow 1 | GRW11 | *Plasmodium relictum* |
| 53 | 0 | 0 | Pipiens | House sparrow 1 | GRW11 | *Plasmodium relictum* |
| 54 | 0 | 0 | Pipiens | House sparrow 1 | GRW11 | *Plasmodium relictum* |
| 55 | 0 | 0 | Pipiens | House sparrow 1 | GRW11 | *Plasmodium relictum* |
| 56 | 0 | 0 | Hybrids | House sparrow 1 | GRW11 | *Plasmodium relictum* |
| 57 | 0 | 0 | Pipiens | House sparrow 1 | GRW11 | *Plasmodium relictum* |
| 58 | 1 | 1 | Pipiens | House sparrow 2 | PADOM01 | *Plasmodium cathemerium* |
| 59 | 1 | 0 | Pipiens | House sparrow 2 | PADOM01 | *Plasmodium cathemerium* |
| 60 | 1 | 0 | Hybrids | House sparrow 2 | PADOM01 | *Plasmodium cathemerium* |
| 61 | 1 | 1 | Hybrids | House sparrow 2 | PADOM01 | *Plasmodium cathemerium* |
| 62 | 1 | 0 | Hybrids | House sparrow 2 | PADOM01 | *Plasmodium cathemerium* |
| 63 | 1 | 1 | Pipiens | House sparrow 2 | PADOM01 | *Plasmodium cathemerium* |
| 64 | 1 | 0 | Hybrids | House sparrow 2 | PADOM01 | *Plasmodium cathemerium* |
| 65 | 1 | 0 | Hybrids | House sparrow 2 | PADOM01 | *Plasmodium cathemerium* |
| 66 | 0 | 0 | Pipiens | House sparrow 2 | PADOM01 | *Plasmodium cathemerium* |
| 67 | 0 | 0 | Pipiens | House sparrow 2 | PADOM01 | *Plasmodium cathemerium* |
| 68 | 1 | 0 | Hybrids | House sparrow 2 | PADOM01 | *Plasmodium cathemerium* |
| 69 | 1 | 0 | Molestus | House sparrow 2 | PADOM01 | *Plasmodium cathemerium* |
| 70 | 1 | 0 | Pipiens | House sparrow 2 | PADOM01 | *Plasmodium cathemerium* |
| 71 | 1 | 1 | Hybrids | House sparrow 2 | PADOM01 | *Plasmodium cathemerium* |
| 72 | 1 | 1 | Pipiens | House sparrow 2 | PADOM01 | *Plasmodium cathemerium* |
| 73 | 1 | 0 | Pipiens | House sparrow 2 | PADOM01 | *Plasmodium cathemerium* |
| 74 | 1 | 1 | Pipiens | House sparrow 2 | PADOM01 | *Plasmodium cathemerium* |
| 75 | 0 | 0 | Hybrids | House sparrow 2 | PADOM01 | *Plasmodium cathemerium* |
| 76 | 0 | 0 | Pipiens | House sparrow 3 | SGS1 | *Plasmodium relictum* |
| 77 | 1 | 0 | Molestus | House sparrow 3 | SGS1 | *Plasmodium relictum* |
| 78 | 0 | 0 | Hybrids | House sparrow 3 | SGS1 | *Plasmodium relictum* |
| 79 | 1 | 0 | Pipiens | House sparrow 3 | SGS1 | *Plasmodium relictum* |
| 80 | 1 | 0 | Hybrids | House sparrow 3 | SGS1 | *Plasmodium relictum* |
| 81 | 0 | 0 | Hybrids | House sparrow 3 | SGS1 | *Plasmodium relictum* |
| 82 | 0 | 0 | Pipiens | House sparrow 3 | SGS1 | *Plasmodium relictum* |
| 83 | 0 | 0 | Pipiens | House sparrow 3 | SGS1 | *Plasmodium relictum* |
| 84 | 0 | 0 | Hybrids | House sparrow 3 | SGS1 | *Plasmodium relictum* |
| 85 | 0 | 0 | Hybrids | House sparrow 3 | SGS1 | *Plasmodium relictum* |
| 86 | 0 | 0 | Hybrids | House sparrow 3 | SGS1 | *Plasmodium relictum* |
| 87 | 1 | 0 | Pipiens | House sparrow 3 | SGS1 | *Plasmodium relictum* |
| 88 | 1 | 0 | Pipiens | House sparrow 3 | SGS1 | *Plasmodium relictum* |
| 89 | 0 | 0 | Hybrids | House sparrow 3 | SGS1 | *Plasmodium relictum* |
| 90 | 0 | 0 | Pipiens | House sparrow 3 | SGS1 | *Plasmodium relictum* |
| 91 | 1 | 0 | Hybrids | House sparrow 3 | SGS1 | *Plasmodium relictum* |
| 92 | 0 | 0 | Hybrids | House sparrow 3 | SGS1 | *Plasmodium relictum* |
| 93 | 1 | 0 | Hybrids | House sparrow 3 | SGS1 | *Plasmodium relictum* |
| 94 | 1 | 0 | Pipiens | House sparrow 3 | SGS1 | *Plasmodium relictum* |
| 95 | 1 | NA | Pipiens | House sparrow 3 | SGS1 | *Plasmodium relictum* |
| 96 | 1 | 0 | Hybrids | House sparrow 3 | SGS1 | *Plasmodium relictum* |
| 97 | 1 | NA | Pipiens | House sparrow 3 | SGS1 | *Plasmodium relictum* |
| 98 | 1 | 0 | Pipiens | House sparrow 2 | PADOM01 | *Plasmodium cathemerium* |
